# Identification of a claudin-low subtype in clear cell renal cell carcinoma with implications for the evaluation of clinical outcomes and treatment efficacy

**DOI:** 10.3389/fimmu.2022.1020729

**Published:** 2022-11-21

**Authors:** Cuijian Zhang, Yifan Li, Jinqin Qian, Zhenpeng Zhu, Cong Huang, Zhisong He, Liqun Zhou, Yanqing Gong

**Affiliations:** ^1^ Department of Urology, Peking University First Hospital, Beijing, China; ^2^ Institute of Urology, Peking University, Beijing, China; ^3^ National Urological Cancer Center, Peking University First Hospital, Beijing, China

**Keywords:** clear cell renal cell carcinoma (ccRCC), claudin, tumor microenvironment, immunity, prognosis, immune checkpoint inhibitor (ICI)

## Abstract

**Background:**

In bladder and breast cancer, the claudin-low subtype is widely identified, revealing a distinct tumor microenvironment (TME) and immunological feature. Although we have previously identified individual claudin members as prognostic biomarkers in clear cell renal cell carcinoma (ccRCC), the existence of an intrinsic claudin-low subtype and its interplay with TME and clinical outcomes remains unclear.

**Methods:**

Transcriptomic and clinical data from The Cancer Genome Atlas (TCGA)- kidney clear cell carcinoma (KIRC) cohort and E-MTAB-1980 were derived as the training and validation cohorts, respectively. In addition, GSE40435, GSE53757, International Cancer Genome Consortium (ICGC) datasets, and RNA-sequencing data from local ccRCC patients were utilized as validation cohorts for claudin clustering based on silhouette scores. Using weighted correlation network analysis (WGCNA) and multiple machine learning algorithms, including least absolute shrinkage and selection operator (LASSO), CoxBoost, and random forest, we constructed a claudin-TME related (CTR) risk signature. Furthermore, the CTR associated genomic characteristics, immunity, and treatment sensitivity were evaluated.

**Results:**

A claudin-low phenotype was identified and associated with an inferior survival and distinct TME and cancer immunity characteristics. Based on its interaction with TME, a risk signature was developed with robust prognostic prediction accuracy. Moreover, we found its association with a claudin-low, stem-like phenotype and advanced clinicopathological features. Intriguingly, it was also effective in kidney chromophobe and renal papillary cell carcinoma. The high CTR group exhibited genomic characteristics similar to those of claudin-low phenotype, including increased chromosomal instability (such as deletions at 9p) and risk genomic alterations (especially *BAP1* and *SETD2*). In addition, a higher abundance of CD8 T cells and overexpression of immune checkpoints, such as LAG3, CTLA4 and PDCD1, were identified in the high CTR group. Notably, ccRCC patients with high CTR were potentially more sensitive to immune checkpoint inhibitors; their counterparts could have more clinical benefits when treated with antiangiogenic drugs, mTOR, or HIF inhibitors.

**Conclusion:**

We comprehensively evaluated the expression features of claudin genes and identified a claudin-low phenotype in ccRCC. In addition, its related signature could robustly predict the prognosis and provide guide for personalizing management strategies.

## 1 Introduction

Renal cell carcinoma (RCC) is the third most prevalent genitourinary cancer worldwide, with an estimated 431,288 new cases diagnosed in 2020 (1). More than 70% of RCC cases are histologically classified as clear cell RCC (ccRCC), and approximately 65% of them are localized and can be treated with surgical resection in the form of partial or radical nephrectomy (2). However, nearly 20–40% of ccRCC patients may experience disease recurrence or develop metastases (3). Multiple prognostic models have been established and clinically verified to improve patient management, such as the UCLA Integrated Staging System (UISS), Leibovich score 2003/2018, VENUSS score, and GRANT score (4). However, these models are mainly based on traditional clinical and pathological variables and present with heterogeneous predictive accuracy according to the pathological characteristics (5). In the meantime, significant breakthroughs have been made in the past two decades, such as introducing vascular endothelial growth factor tyrosine kinase inhibitors (VEGFR TKIs), mammalian target of rapamycin (mTOR) inhibitors, and particularly immune checkpoint inhibitors (ICIs) to better manage patients with ccRCC. However, even when applying the most effective immune combination therapy, clinical benefits are limited to a certain section of patients (6). Thus, there is an urgent need to develop novel and powerful prognostic prediction biomarkers for both risk and treatment stratification.

Claudins are the backbone of the tight junction complex, which includes a group of proteins 20–34 kDa in size and a structure similar to that of a short cytoplasmic N-terminal region, two extracellular loops formed by four transmembrane domains, and a cytoplasmic C-terminal tail (7). To date, 24 claudin family members, which are commonly downregulated in tumor tissues, have been identified; however, their roles in the regulation of the development of different types of cancer are heterogeneous (8). In a previous study, we found that claudin 7 is a tumor suppressor in ccRCC, and hypermethylation of its promoter or its downregulation facilitates epithelial-mesenchymal transition (EMT) and tumor progression (9). Other claudin members, including claudin-2 (10), claudin-4 (11), claudin-5 (12), and claudin-8 (13), have been investigated in ccRCC as an individual prognostic biomarker, respectively. It is noteworthy that all previous studies were focused on a single member of claudin; thus, little is known about the comprehensive expression profile of claudin family members in ccRCC.

Meanwhile, claudin-low subtype has been widely recognized as a novel intrinsic subtype in both breast and bladder cancer, exhibiting aggressive, distinct biological and clinical behaviors (14). Previous studies have depicted its correlation with EMT and stemness in the tumor, revealing its origin and evolution (15). More importantly, increasing evidence supports the interaction between claudin phenotype and the immune profile of tumors. For instance, bladder or breast tumors with subtype classification based on claudin expression showed distinct immune features, characterized by different levels of tumor-infiltrating immune cells and expression of immune checkpoints such as programmed death-ligand 1 (PD-L1) (16, 17). These features may further contribute to a better response to immune checkpoint inhibitors (ICIs) (18). However, to the best of our knowledge, there was no study on the claudin-low phenotype in ccRCC and whether there is a correlation between claudin, cancer immunity and prognosis in patients with ccRCC remains elusive.

In this study, we sought to analyze the comprehensive landscape of the expression of claudin family members in ccRCC and mainly focused on the interaction between claudin expression features and immunity. Subsequently, a prognostic prediction signature was developed based on candidate claudin-immune genes, and its correlation with precision medicine including targeted therapy and ICIs was explored.

## 2 Materials and methods

### 2.1 Study design

**Figure 1:**
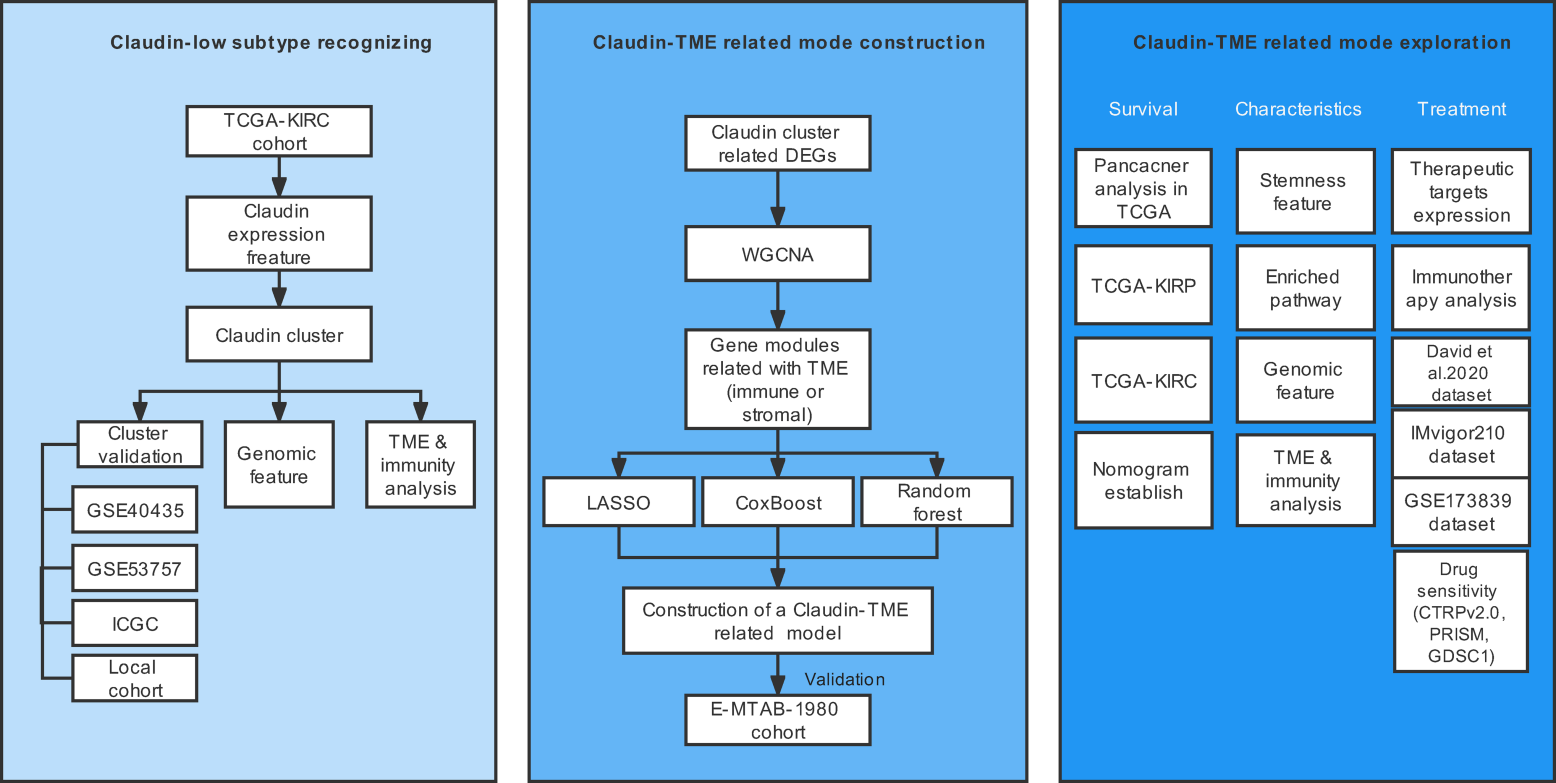
Overview of the study design. TME: tumor microenvironment.

The schematic workflow is shown in **Figure 1**.

### 2.2 Data retrieval and preprocessing

The RNA-sequencing (RNA-seq) and clinical data of 530 ccRCC samples (and 72 adjacent nontumor tissues) were downloaded from The Cancer Genome Atlas (TCGA)- kidney clear cell carcinoma (KIRC) cohort *via* the cbioportal website (https://www.cbioportal.org). The mRNA expression profile and clinical data of E-MTAB-1980 with 101 samples were downloaded from the ArrayExpress database (https://www.ebi.ac.uk/arrayexpress/) and used as the validation cohort for survival analysis. GSE40435 (tumor vs. normal tissue: 101 vs. 101), GSE53757 (72 vs. 72), and the International Cancer Genome Consortium (ICGC) (91 vs. 45) datasets were used as validation cohorts for claudin clustering and identify the differences in differentially expressed genes between tumor and normal kidney tissues.

### 2.3 RNA sequencing in the local ccRCC patients

Tumor and matched normal tissues were collected from 20 ccRCC patients in our local cohort to perform RNA-seq. This study was approved by the Biomedical Research Ethics Committee of Peking University First Hospital (approval no. 2015-977) and written informed consent was obtained from all the patients. Clinicopathological data of the 20 enrolled patients are presented in **Supplementary Table 1**. Before RNA extraction, the tissue was evaluated for tumor cell content and percentage, and only those with a tumor purity of at least 20% based on histopathological analysis were eligible for RNA extraction and sequencing. Total RNA from each sample was collected using a FastPure^®^ Cell/Tissue Total RNA Isolation Kit V2 (Vazyme, Jiangsu, China), and the RNA concentration and RNA integrity number (RIN) were measured using a Qubit (Thermo Fisher Scientific, MA, United States) and an Agilent 2100 bioanalyzer (Agilent Technologies, CA, United States), respectively. Library construction was performed using the NEBNext^®^ Ultra™ RNA Library Prep Kit for Illumina^®^ Kit (NEB, MA, United States) and sequenced on the Illumina Novaseq-6000 system (Illumina, MA, United States).

### 2.4 Cluster of claudin expression profile in ccRCC

We calculated the similarity of claudin family gene expression in ccRCC samples in the TCGA-KIRC database using R package “factoextra” and the silhouette scores were obtained based on the assigned clusters. The optimal number of clusters in each cohort was determined using the silhouette width.

### 2.5 Differentially expressed genes and weighted gene co-expression network analysis

DEGs with a threshold of log_2_(FoldChange) > 0.585 and adjusted p< 0.05 were identified using the R package “limma”. DEGs between claudin clusters were collected for WGCNA using the R package “WGCNA”. The appropriate power value was determined when the scale independence was > 0.85 with a relatively higher connectivity degree. When the scale independence was > 0.85 and the connectivity degree was relatively higher, the appropriate power value was determined. Genes were then sorted into several gene modules based on topological overlap matrix (TOM)-based dissimilarities. Finally, the dynamic modules were merged according to a cut-off value of 0.25 and five modules were obtained. Gene modules with correlation coefficient > 0.5 with immune and/or stromal scores, were recognized as tumor microenvironment (TME)-related gene modules.

### 2.6 Stromal and immune score analysis

Estimation of stromal and immune cells in malignant tumor tissues using expression data (ESTIMATE) algorithm depicts the level of tumor-infiltrating immune cells, stromal cells, and tumor purity in the form of immune score, stromal score, and ESTIMATE score, respectively (19). The stromal and immune scores of each sample were calculated using the R package “ESTIMATE”.

### 2.7 Construction of a novel claudin-TME related prognostic prediction signature

Hub genes screened out in the TME gene module determined by WGCNA were then inputted into three machine learning algorithms, including least absolute shrinkage and selection operator (LASSO) regression analysis (R package “glmnet,” version 4.1-4), CoxBoost (R package “CoxBoost,” version 1.5) and random forest (R package “randomForestSRC,” version 3.1.0). We chose to use three algorithms as opposed to only one to reduce the risk of bias, and the overlapping hub genes shared by all of them were selected to construct a risk signature. The CTR prognostic prediction was developed using the following formula:


$$CTR\;score=\sum_{i}^{}\text{Coefficient of }\left(\text{Gene }i\right)\;\times \text{ Expression of }\left(\text{Gene }i\right)\;$$

The patients in each cohort were dichotomized into high- and low-risk groups based on the median CTR score. A time-dependent receiver operating characteristic (ROC) curve was applied to analyze the predictive accuracy of CTR signature in the training and validation cohorts.

### 2.8 TME and immune profile analysis

Tumor-infiltrating immune cell profiles were comprehensively analyzed using seven different algorithms, including CIBERSOFTX (20), Microenvironment Cell Populations (MCP)-counter (21), tumor immune estimation resource (TIMER2.0) (22), and xCell (23). The seven steps involved in the cancer-immune cycle in each sample, starting from the release of cancer cell antigens to the killing of cancer cells, was evaluated using single-sample gene set enrichment analysis (ssGSEA) (24).

### 2.9 Stemness feature analysis

Tumor stem cell-like features (stemness) were indicated as the mRNA expression-based stemness index (mRNAsi), which was calculated using the method described by Malta et al. (25)

### 2.10 Genomic profile analysis

Genetic alterations were analyzed using the “maftools” package in the TCGA-KIRC database. Comparison of the prevalence of genomic alterations in certain genes was conducted between high and low CTR groups using Fisher’s exact test, and only those genes with a prevalence of over 3% in at least one group were included.

### 2.11 Comparison with other risk models of ccRCC

Thirteen previously defined risk models for ccRCC were identified through the PubMed database (26–38), and the risk score for each model was determined based on the algorithm provided in the corresponding published study. The details are listed in **Supplementary Table 2**. Differences in area under the ROC curve (AUC) for survival at 1–5 years and the concordance index (C-index) were compared between the CTR score and these 13 risk models in the TCGA-KIRC dataset based on a bootstrap resampling method.

### 2.12 Nomogram construction

Univariate and multivariate Cox regression analyses were performed on clinicopathological variables, including age, sex, neoplasm histologic grade, neoplasm disease stage, tumor, nodes, and metastases (TNM) stage, and risk score in the TCGA-KIRC database. Variables were integrated to establish a nomogram using the “rms” R package.

### 2.13 Treatment sensitivity analysis

#### 2.13.1 Evaluation of the expression of therapeutic targets in ccRCC

A comprehensive evaluation of the difference in the expression level of the target of Food and Drug Administration (FDA)-approved drugs, including ICIs (anti-CTLA4 and anti-PD-1/PD-L1), antiangiogenic therapy (bevacizumab, and targeted therapies including sunitinib, pazopanib, axitinib, cabozantinib, lenvatinib, tivozanib, and sorafenib), mTOR inhibitors (everolimus and temsirolimus), and hypoxia-inducible factor 2α (HIF2A) inhibitor (belzutifan) between the high- and low-risk groups was performed. Matched drug-target information was retrieved from the DrugBank website (https://go.drugbank.com/). The Cancer Dependency Map (DepMap) portal (https://depmap.org/portal/) was used to evaluate the drug sensitivity of therapeutic targets from three databases (CTRPv2.0, PRISM, and GDSC1). Lower AUC values for the agents indicated a higher drug sensitivity.

#### 2.13.2 ICI

The clinical and gene expression profiles of ccRCC patients treated with nivolumab in the CheckMate 025, 010, and 009 trials were derived from the dataset published by David et al. (39) The clinical response and genomic data from the IMvigor210 cohort (28) and GSE173839 dataset (40) were analyzed for differences in clinical benefits from ICI based on stratification using the CTR score. The criteria for treatment responses were defined as follows: CR: complete response, PR: partial response, SD: stable disease, and PD: progressive disease.

### 2.14 Statistical analysis

All statistical analyses were performed using the R software (version 4.1.2). Kaplan-Meier curves and log-rank tests were used to analyze the survival differences between categorical variables in each cohort. Spearman correlation coefficients were analyzed to explore the correlations between different variables. Chi-squared test or Fisher’s exact test were used to compare the differences in categorical variables. Statistical significance was defined as a two-sided p value< 0.05.

## 3 Results

### 3.1 Claudin expression pattern in ccRCC

**Figure 2:**
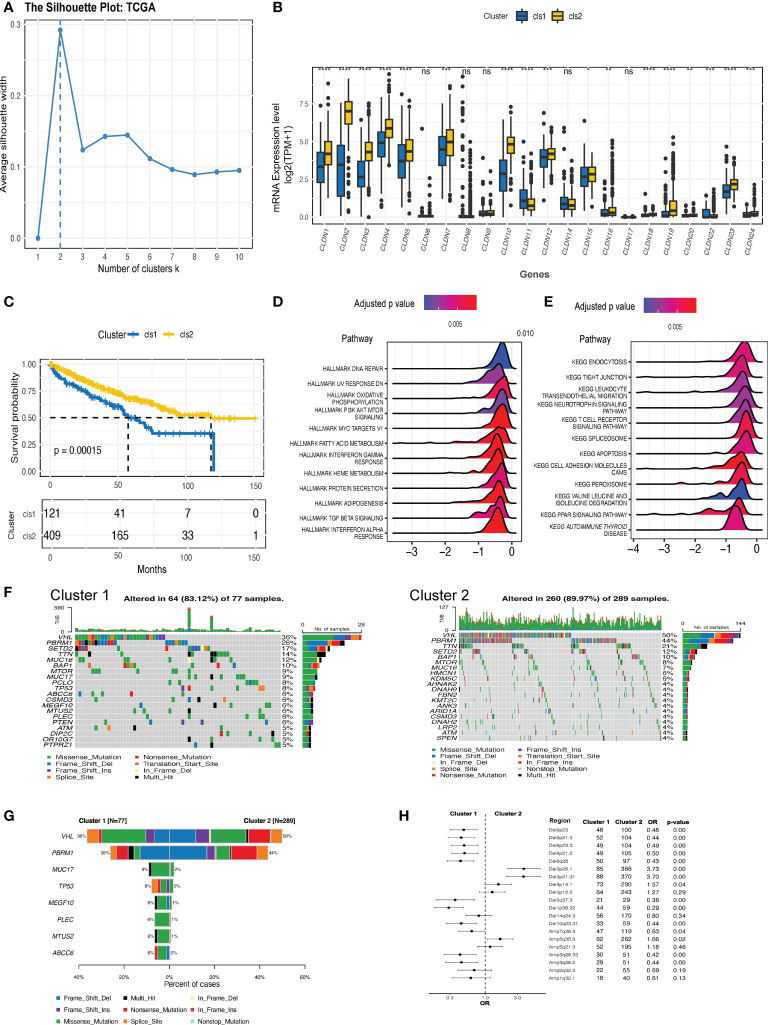
The expression clusters of claudin genes in ccRCC. **(A)** Silhouette clustering analysis in The Cancer Genome Atlas-kidney clear cell carcinoma (TCGA-KIRC) cohort. **(B)** The difference in the expression level of each claudin gene between clusters 1 and 2. **(C)** Kaplan–Meier estimates of the difference in the overall survival between cluster 1 and cluster 2. Hallmark **(D)** and Kyoto Encyclopedia of Genes and Genomes (KEGG) **(E)** pathways analysis revealed the pathways significantly enriched in the differentially expressed genes (DEGs) between the two clusters. **(F)** Oncoprints of the top 20 prevalent genes in cluster 1 (left) and cluster 2 (right). **(G)** Genes with significantly differed prevalence in cluster 1 or cluster 2. Only genes with a prevalence of over 3% in at least one cluster were analyzed. **(H)** Difference in the prevalence of copy number variants (CNV) between clusters 1 and 2. OR value below 1 is indicated as more prevalent in cluster 1. Del, deletion; Amp, amplification; OR, odds ratio; TPM, transcript per million. *p<0.05, **p<0.01, ***p<0.001, ****p<0.0001. ns, not significant.

An overview of the expression features of all the claudin family genes in three kinds of kidney cancer revealed that nearly all the genes were downregulated in ccRCC tumors (**Supplementary Figure 1A**), with the exception of claudin 18 in ccRCC. However, the predictive function of each claudin member was inconsistent (**Supplementary Figure 1B**), highlighting the need for integrative research. As more claudin genes influenced patient survival in ccRCC, we focused our investigational study on this histological type. Based on the comprehensive expression profiles of claudin family genes, we identified two distinct claudin expression clusters in the TCGA-KIRC dataset (**Figure 2A**). To validate whether the clustering numbers were comprehensively optimal, we utilized the GSE40435, GSE53757, and ICGC datasets, and local ccRCC samples to evaluate the similarity of the expression of claudin family members and confirmed that clustering by two had the highest average silhouette width in all three datasets (**Supplementary Figure 2A**). Interestingly, a comparison of the difference in each claudin gene between these two clusters showed that cluster 1 also exhibited a notable claudin-low feature, characterized by an overall lower expression level of claudin genes (**Figure 2B**). Twenty-two percent (121/530) of ccRCC patients belonged to cluster 1, which had significantly inferior overall survival (**Figure 2C**), and more patients with advanced neoplasm histologic grades and/or stages were present in this cluster (**Supplementary Table 3**). This claudin-low feature in cluster 1 was also validated in other datasets and samples from local ccRCC patients (**Supplementary Figure 2B**). Altogether, 2806 DEGs were identified between these two clusters (**Supplementary Table 4**), which were mainly enriched in multiple cell signaling, transduction, metabolism, and particularly the immune regulation pathways, including leukocyte transendothelial migration and T cell receptor signaling pathways (**Figures 2D, E**). Two clusters revealed distinct genomic features (**Figures 2F, G**): *VHL* and *PBRM1* were significantly more prevalent in cluster 2; whereas genes including *MUC17*, *TP53*, *MEGF10* were more mutated in cluster 1. In addition, cluster 1 showed significantly increased chromosomal instability, as evidenced by growing deletions at 9p23, 9p21, 8p23, and 8p21, as well as amplifications at 3q26.33 and 3q36.2 (**Figure 2H**).

### 3.2 Claudin expression cluster and tumor microenvironment in ccRCC

**Figure 3:**
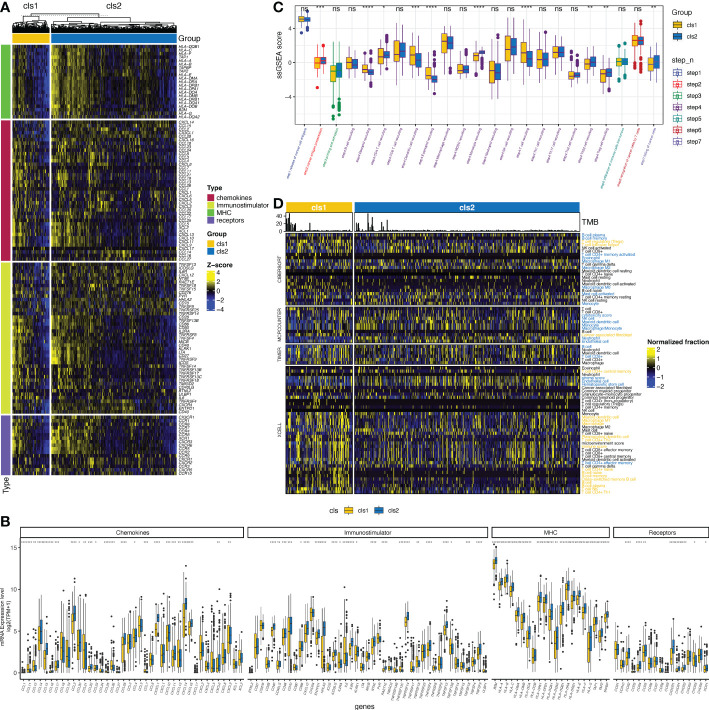
Claudin expression cluster and tumor microenvironment in ccRCC. **(A)** Difference in the expression of regulators of tumor immunology, including chemokines and their receptors, major histocompatibility complex (MHC), and immunostimulators between the two clusters in The Cancer Genome Atlas (TCGA)- kidney clear cell carcinoma (TCGA-KIRC) cohort. **(B)** Bar plot shows the difference of the expression of regulators of tumor immunology between two clusters. **(C)** Differences in the activity of seven steps of the cancer-immunity cycle between clusters. **(D)** Analysis of the difference in the tumor-infiltrated immune cells (TIICs) between the two clusters using multiple computational algorithms, including CIBERSOFT, MPCOUNTER, TIMER, and XCELL. TIICs with yellow color were significantly more enriched in cluster 1, whereas those with blue color were more enriched in cluster 2 (p< 0.05). *p< 0.05, **p< 0.01, ***p< 0.001, ****p< 0.0001, ns, not significant; TPM, transcript per million; ssGSEA, single-sample gene set enrichment analysis.

As previous studies have suggested a correlation between the claudin-low phenotype and increasing levels of immune and stromal cell infiltration in breast cancer (41), we then evaluated the interaction between claudin expression features and TME profiles in ccRCC. The claudin-low related cluster (cluster 1) was associated with changes in multiple immunomodulators (**Figures 3A, B**), with significant downregulation of major histocompatibility complex (MHC) molecules, which revealed the association between claudin-low feature and blockade of the antigen presentation process (**Figures 3A, B**). Chemokines and their receptors, as well as immune stimulators, were also heterogeneous between these two clusters, and most of them were downregulated in cluster 1. As shown in **Figure 3C**, there was no significant difference in the release of cancer cell antigens (step 1), but after the cancer antigen presentation process (step 2), the two clusters began to differ in the activity of cancer immunity. Cancer antigen presentation was downregulated in cluster 1, as well as in CD4 T cells, Th22 cells, monocytes, and Treg cells (step 3). However, cluster 1 showed significantly upregulated levels of T cells, dendritic cells, eosinophils, and basophils. Finally, all these differences contributed to a reduced killing of the cancer cells (step 7) in cluster 1. Furthermore, by applying multiple algorithms, we found a distinct feature in tumor-infiltrating immune cells (TIICs) between these two clusters (**Figure 3D**), including an increase in Treg cells but a decrease in B cells. Even though different algorithms gave discordant results, there was a concordant difference in the levels of B cells, macrophages, and CD4 T cells between the two clusters.

### 3.3 Construction of a claudin-TME related risk signature

**Figure 4:**
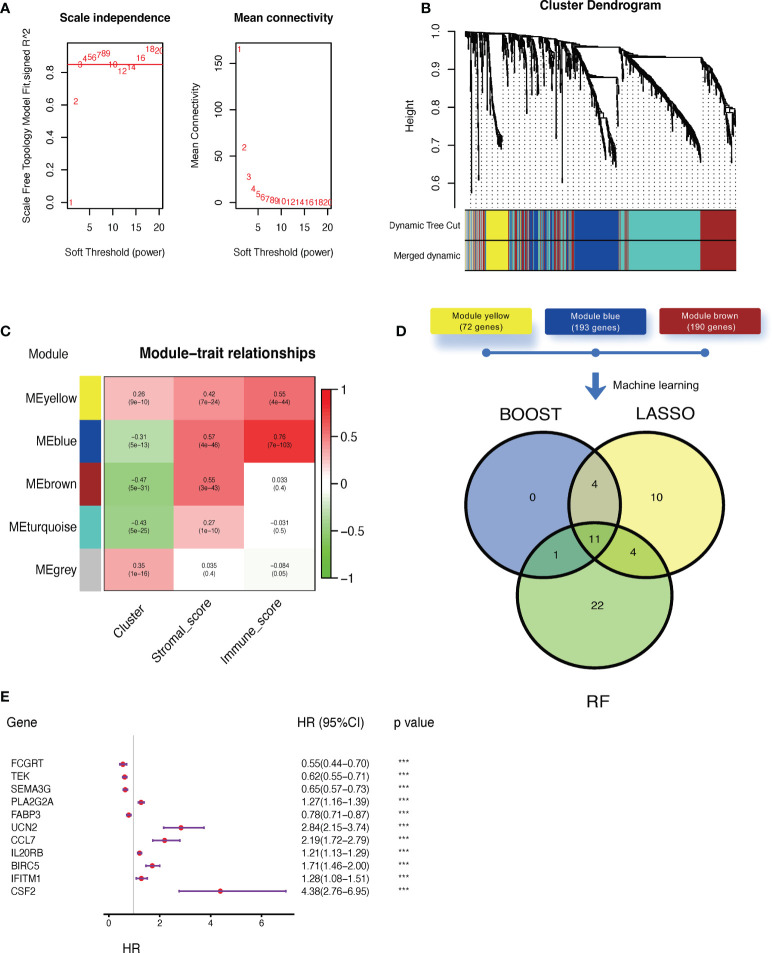
Construction of a claudin-TME related (CTR) risk signature. **(A)** Analysis of the scale-free fit index (left) and mean connectivity (right) for different soft-thresholding (power) values (numbers colored with red). **(B)** Gene clustered dendrogram based on dissimilarity measure (1-topological overlap matrix (TOM)) with defined module colors. **(C)** Heatmap of the correlations between module genes and cluster, stromal, or immune scores. **(D)** Venn plot showing the overlapping genes selected by three machine learning algorithms: CoxBoost, least absolute shrinkage and selection operator (LASSO), and random forest (RF). **(E)** Forest plot of hazard ratios for selected key genes in the Cancer Genome Atlas -kidney clear cell carcinoma (TCGA-KIRC) dataset. ***p< 0.001. HR: hazard ratio. 95% CI: 95% confidence interval.

Next, we applied WGCNA to distinguish co-expressed gene modules within cluster-related genes. After 10 was determined as the optimal soft threshold (**Figure 4A**), five co-expressed gene modules were identified (**Figure 4B**). As depicted in **Figure 4C**, module yellow (containing 72 genes), module blue (193 genes), and module brown (190 genes) were strongly correlated with stromal and/or immune scores. Genes within these three modules were then selected to be used in constructing the model (**Supplementary Table 5**). Then, three algorithms, including CoxBoost, LASSO, and random forest (RF) were applied and the c-index was 0.77, 0.72 and 0.82, respectively. Even though they all had satisfied prediction capacity, we integrated them to eliminate the any potential bias in a single machine learning algorithm. Finally, 11 key genes were screened out by all three algorithms (**Figure 4D**;, **Supplementary Table 6**), of which only four were suggested as tumor suppressor genes while the rest were associated with worse outcomes in ccRCC (**Figure 4E**). The final claudin-TME risk signature was as follows: CTR score = 0.3518×EXP_CSF2_ − 0.3948×EXP_FCGRT_ − 0.3519×EXP_TEK_ − 0.0502×EXP_SEMA3G_ + 0.0775×EXP_PLA2G2A_ − 0.0513×EXP_FABP3_ + 0.2117×EXP_UCN2_ + 0.2910×EXP_CCL7_ + 0.0608×EXP_IL20RB_ + 0.0977×EXP_BIRC5_ + 0.3381×EXP_IFITM1_.

### 3.4 Evaluation and validation of the prognostic predictive accuracy of CTR signature

**Figure 5:**
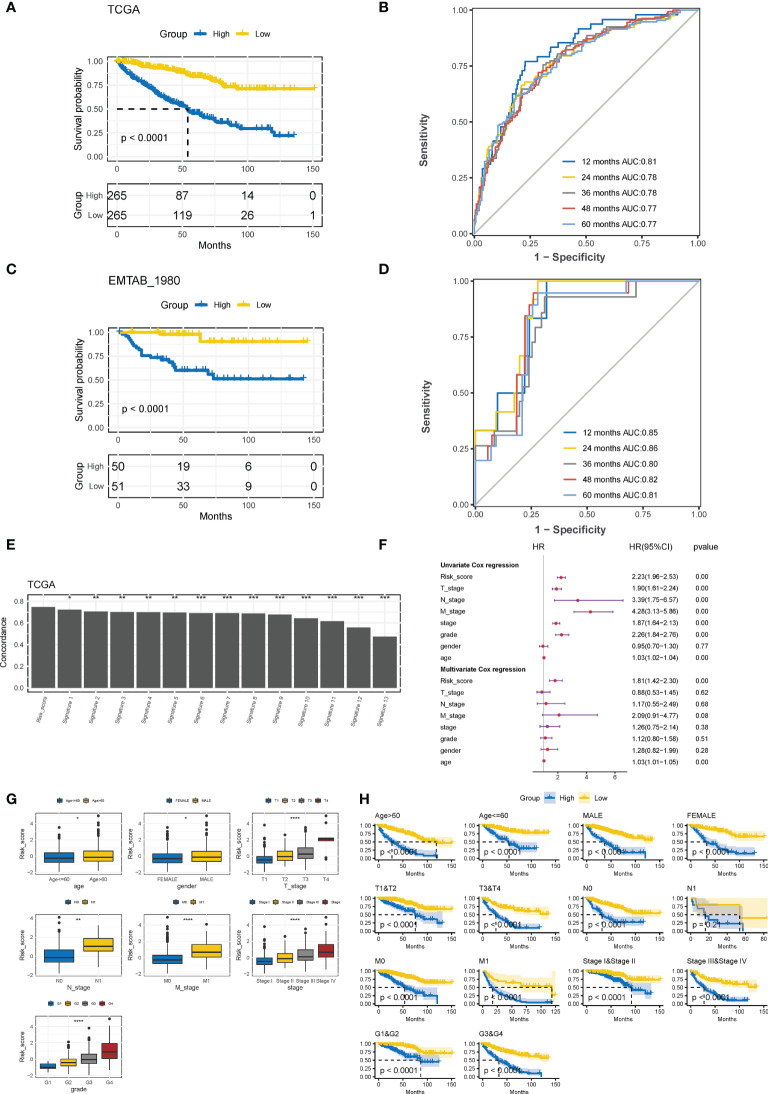
Evaluation and validation of prognostic predictive accuracy of CTR signature. **(A)** Kaplan–Meier survival analysis of the difference in the overall survival (OS) between ccRCC patients with high and low claudin-TME related (CTR) score in The Cancer Genome Atlas-kidney clear cell carcinoma (TCGA-KIRC) training cohort. Patients were dichotomized into high or low CTR groups based on the median CTR score. **(B)** Time-dependent receiver operating characteristic (ROC) curves at 1–5 years in the training cohort. **(C)** Kaplan–Meier estimates of the difference in the OS between ccRCC patients with high and low CTR scores in the validation cohort (E-MATAB-1980). Patients were also dichotomized into high or low CTR groups based on the median CTR score. **(D)** Time-dependent area under the ROC curve (AUC) value in the validation cohort. **(E)** Difference in the concordance index (C-index) between the Clinical Risk Groups (CTR) score and other previously reported prognostic models in TCGA-KIRC. **(F)** Forest plot of hazard ratios for CTR score and clinicopathologic variables in the TCGA-KIRC dataset. **(G)** Difference in the distribution of CTR scores between patients with different clinicopathologic variables. **(H)** Kaplan–Meier survival analysis of the CTR score stratified by different clinicopathologic feature. *p< 0.05, **p< 0.01, ***p< 0.001, ****p< 0.0001. CTR score, claudin-TME related risk score; AUC, area under curve; T, Tumor stage; N node stage; M, metastasis stage; Grade, Neoplasm Histologic Grade; Stage, Neoplasm Disease Stage.

Based on the median CTR score, ccRCC patients in the training cohort were dichotomized into high- or low-risk groups, and patients with high-risk scores had significantly inferior clinical outcomes (**Figure 5A**). The AUC of the CTR score for predicting survival at 1-, 2-, 3-, 4-, and 5- year was 0.81, 0.78, 0.78, 0.77, and 0.77, respectively (**Figure 5B**). The prognostic predictive accuracy of the CTR signature was also verified in the validation cohort (**Figure 5C**), which showed a robust predictive capacity (AUC values were all above 0.80) for survival at 1–5 year (**Figure 5D**). Moreover, when compared to previously published ccRCC prognostic models, we discovered that the CTR signature outperformed them in prognostic prediction of the TCGA-KIRC cohort with a significantly greater C-index (**Figure 5E**). Meanwhile, combined univariate and multivariate Cox regression analyses showed that the CTR score was the only significant independent risk factor for prediction survival in the TCGA-KIRC dataset (**Figure 5F**). Interestingly, male patients, those with advanced disease stages, and those > 60 years of age had significantly higher CTR scores (**Figure 5G**). In stratification by a variety of clinicopathological variables, the CTR score consistently distinguished ccRCC patients with a poor prognosis (**Figure 5H**). Notably, even among patients with early stage disease, the CTR score was still able to identify those with worse outcomes.

### 3.5 Exploration of CTR score in pan-cancer

**Figure 6:**
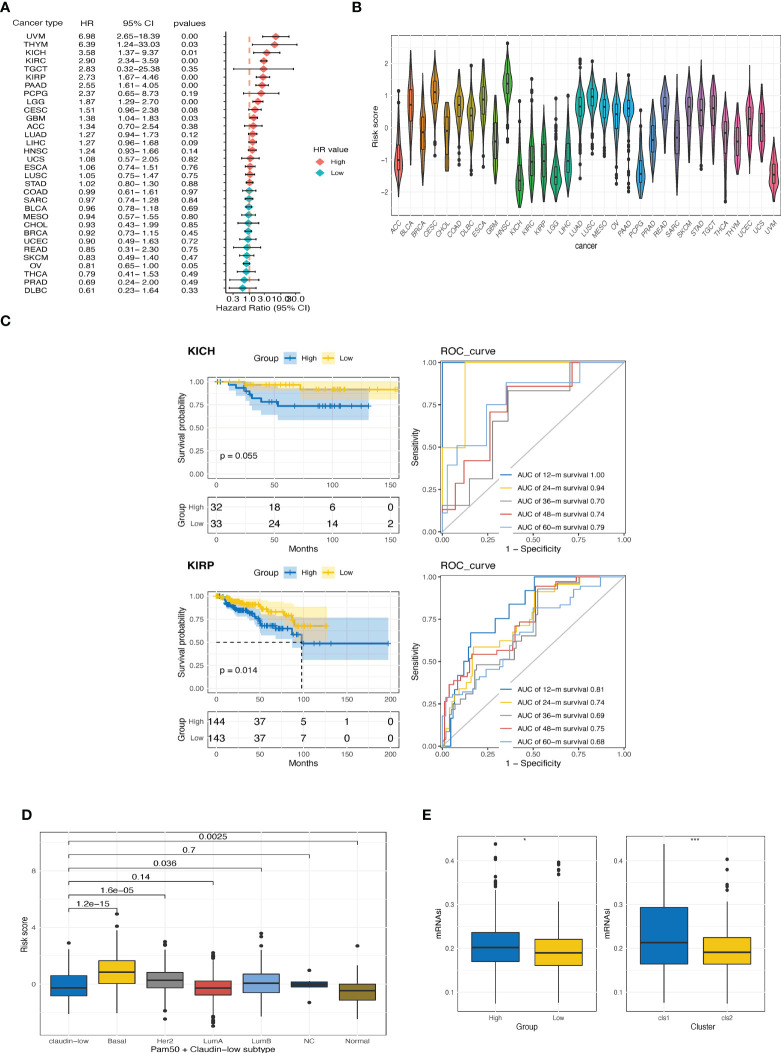
Exploration of CTR score in pan-cancer. **(A)** Forest plot of hazard ratios (HR) for the claudin-TME related (CTR) score in pan-cancers datasets from The Cancer Genome Atlas (TCGA) cohort. **(B)** Distribution of CTR scores among pan-cancers. **(C)** Kaplan–Meier curves and time-dependent area under the curve (AUC) value in the TCGA-kidney chromophobe (KICH) (upper) and TCGA-kidney renal papillary cell carcinoma (KIRP) (bottom) cohorts. **(D)** Difference in CTR scores between claudin-low and other subtypes of breast cancer in the METABRIC cohort. **(E)** Difference in the mRNA expression-based stemness index (mRNAsi) score between high and low CTR groups or between two clusters in the TCGA-kidney renal clear cell carcinoma (KIRC) cohorts. *p<0.05, ***p<0.001. KIRC, kidney renal clear cell carcinoma; KIRP, Kidney renal papillary cell carcinoma; KICH, kidney chromophobe.

We then investigated the prognostic value of the CTR score in pan-cancer datasets from TCGA. Higher CTR scores also indicated inferior outcomes in seven cancer types other than ccRCC, including kidney chromophobe (KICH), kidney renal papillary cell carcinoma (KIRP), uveal melanoma (UVM), thymoma (THYM), pancreatic adenocarcinoma (PAAD), brain low-grade glioma (LGG), and glioblastoma (GBM) (**Figure 6A**). In addition, kidney cancers, regardless of the histological type, had the lowest CTR scores when compared to other cancer types in the pan-cancer dataset (**Figure 6B**). We also evaluated the prediction accuracy of the CTR score for KIRP and KIRC to provide a deeper understanding of the prognostic prediction capacity of these two histological types (**Figure 6C**). The results revealed that the CTR score had a robust prediction accuracy in patients with kidney cancer, regardless of the histologic subtype. The claudin-low phenotype has been primarily explored in breast cancer; hence, we compared the distribution of CTR scores between claudin-low and other phenotypes in breast cancer (The Molecular Taxonomy of Breast Cancer International Consortium (METABRIC) dataset, **Figure 6D**). The lowest CTR score was found in the claudin-low subtype, especially when compared to the basal-like, human epidermal growth factor receptor 2 (HER2)-positive, and luminal B subtypes. Furthermore, the high CTR group had significantly higher mRNA expression-based stemness index (mRNAsi) scores, which indicated a stem cell-like feature in this group (**Figure 6E**).

### 3.6 Enrichment analysis of CTR score

**Figure 7:**
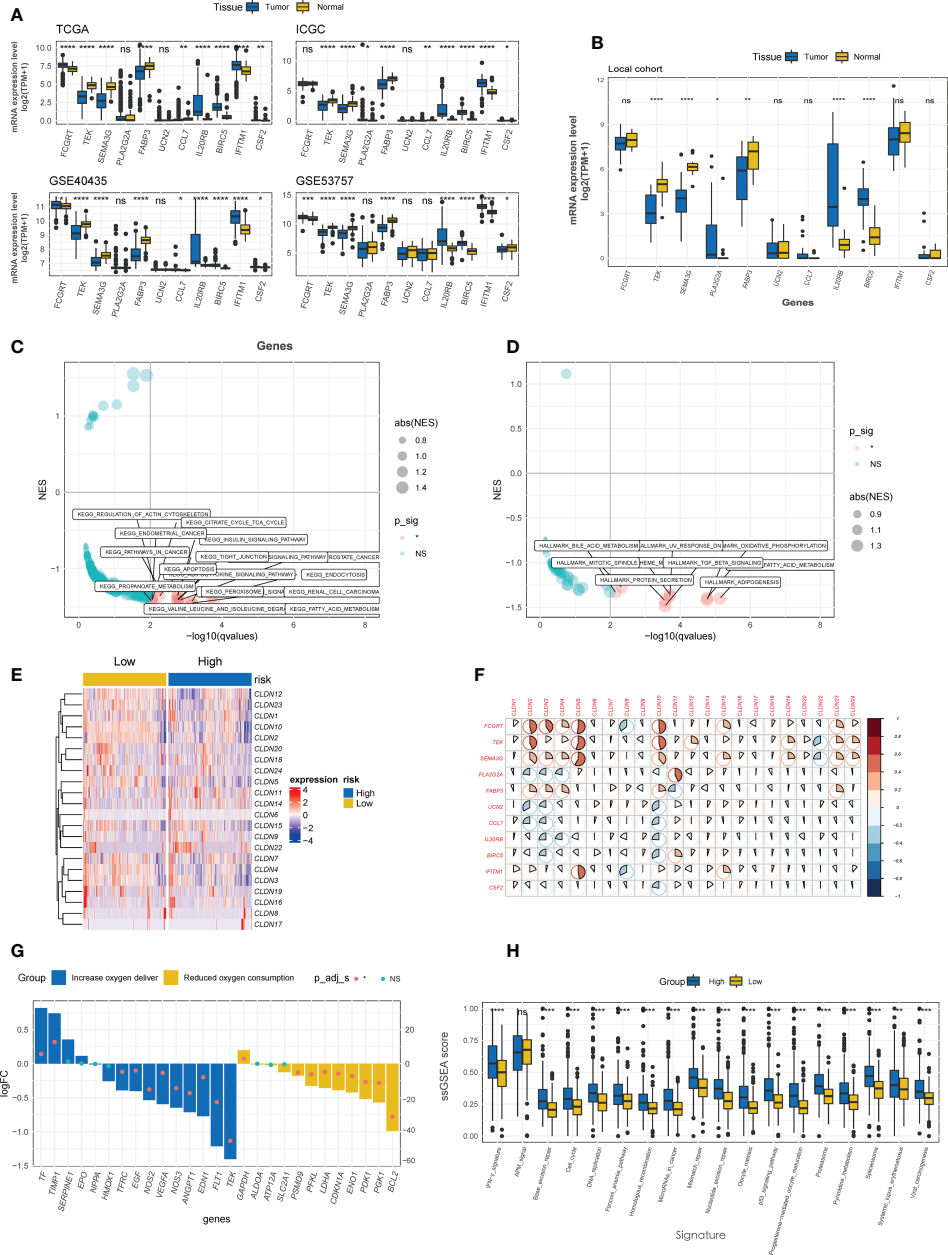
Enrichment analysis of CTR score. **(A)** Comparison of the expression difference of eleven claudin-TME related (CTR) score-related genes between tumor and normal tissues in TCGA, GSE40435, GSE53757 and International Cancer Genome Consortium (ICGC) datasets. **(B)** Difference in the expression level of 11 CTR score-related genes between tumor and normal tissues in local ccRCC patients. Kyoto Encyclopedia of Genes and Genomes (KEGG) **(C)** and Hallmark **(D)** pathways analysis revealed that the pathways were significantly enriched in the differentially expressed genes (DEGs) between high and low CTR groups in the TCGA-KIRC cohort. Only the significantly enriched pathways are shown in the figure. **(E)** Heatmap of the expression of claudin genes in high and low CTR groups in the Cancer Genome Atlas-kidney clear cell carcinoma (TCGA-KIRC) cohort. **(F)** Correlation between CTR score related genes and claudin family genes. **(G)** Expression changes (high vs. low CTR groups) of target genes involved in the hypoxia-inducible factor-1 (HIF-1) KEGG pathway. **(H)** Difference in the enrichment scores of immunotherapy-predicted pathways between high and low CTR groups. *p< 0.05, **p< 0.01, ***p< 0.001, ****p< 0.0001, ns, not significant; TPM, transcript per million; ssGSEA, single-sample gene set enrichment analysis; NES, normalized enrichment score.

Four CTR score-related genes that associated with improved survival were downregulated in kidney tumor tissues relative to normal tissues in both public datasets (**Figure 7A**) and local ccRCC samples (**Figure 7B**), with Fc gamma receptor and transporter (FCGRT) being the sole exception. In contrast, risk genes, except urocortin-2 (UCN2), were significantly upregulated in tumor tissues (**Figures 7A, B**). Moreover, 338 upregulated and 1716 downregulated DEGs were identified between the high and low CTR groups, and these DEGs were significantly enriched in cell metabolism (such as fatty acid metabolism), immune-related pathways (cytokine and transforming growth factor-β (TGF-β)), and cell-cell adhesion and tight junction pathways (tight junction, **Figures 7C, D**). The “claudin-low” characteristic of the high-risk groups was identical to the aforementioned cluster 1, with a generally lower expression level of claudin family genes (**Figure 7E**). Intriguingly, the expression of the four tumor suppressor genes (FCGRT, TEK, semaphorin 3G (SEMA3G), and fatty acid binding protein 3 (FABP3)) was significantly positively correlated with that of claudin genes, whereas the risk genes were shown to be negatively correlated with claudin genes, particularly claudins 2, 3, 4, 5, and 10 (**Figure 7F**). The majority of genes involved in increasing oxygen delivery and reducing oxygen consumption were downregulated in the high CTR group. Contrarily, the high CTR group had significantly higher enrichment scores in nearly all the immunotherapy-positive pathways (**Figure 7H**).

### 3.7 Genomic characteristics related to the CTR score

**Figure 8:**
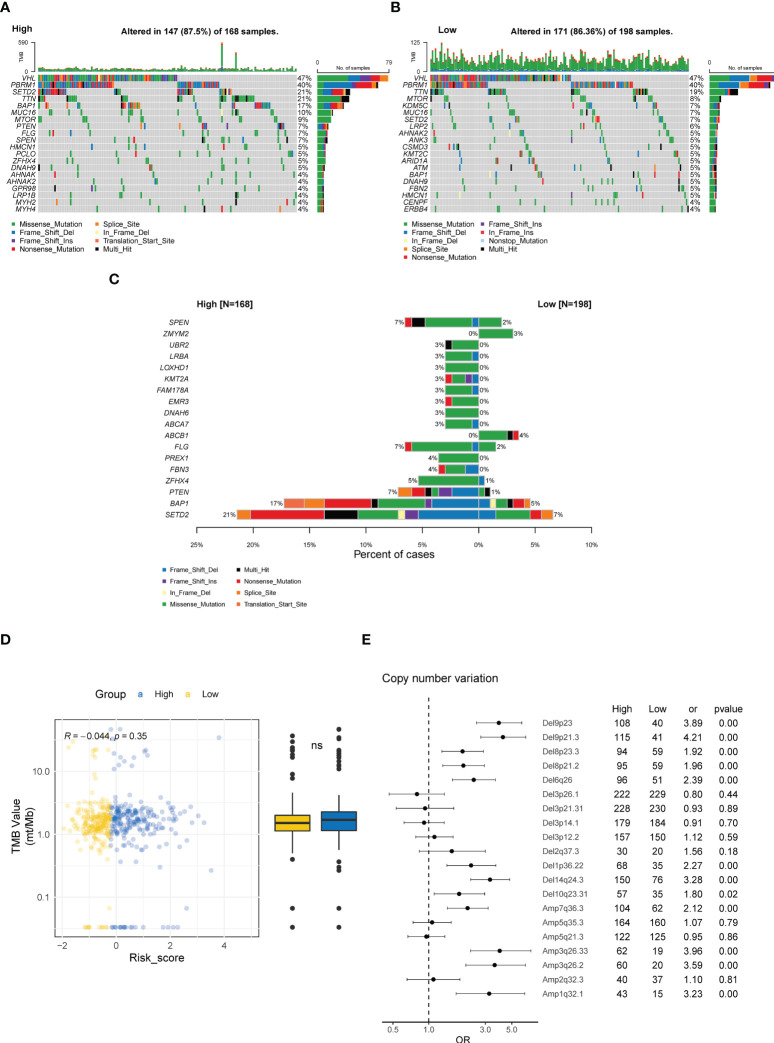
Genomic characteristics related to CTR score. Oncoplots of the top 20 altered genes in the high **(A)** and low CTR groups **(B)**. **(C)** Genes with significantly differed prevalence between the high and low CTR groups. **(D)** Difference in the tumor mutational burden (TMB) level between the high and low CTR groups. **(E)** Difference in the prevalence of copy number variations (CNV) between the high and low CTR groups. Odds ratio (OR) > 1.0 indicated a higher prevalence in the high CTR group; conversely, OR< 1.0 indicated that the event was more prevalent in the low CTR group. TMB, tumor mutation burden; CNV, copy number variant; Del, deletion; Amp, amplification; ns, not significant.

ccRCC tumors are characterized by distinct genomic features, especially the high prevalence of genomic alterations (GAs) in chromosome 3p (42). Hence, we investigated the differences in genomic profiling between the high- and low-risk groups. As depicted in the oncoprint plots in **Figure 8A**, the high and low CTR groups shared genomic features in highly prevalent genes, specifically *VHL*, *PBRM1*, and *TTN* (**Figures 8A, B**). However, distinct differences in the prevalence of altered genes were identified between these two groups, and genes with a significantly higher prevalence were discovered in the high CTR group, including *SETD2*, *BAP1*, *PTEN* and *SPEN* (**Figure 8C**). We did not observe a significant difference in tumor mutational burden (TMB) levels between the two groups (**Figure 8D**). More interestingly, significantly more copy number variations (CNV) were identified in the high CTR group, including a distinguishing prevalence of deletions at 9p23, 9p21.3, 8p23.3, 8p21.2, 6q26, 10q23.31, and amplifications at 3q26.33, 3q26.2 and 1q31.1 (**Figure 8E**).

### 3.8 Immune phenotypes and tumor microenvironment related to the CTR score

**Figure 9:**
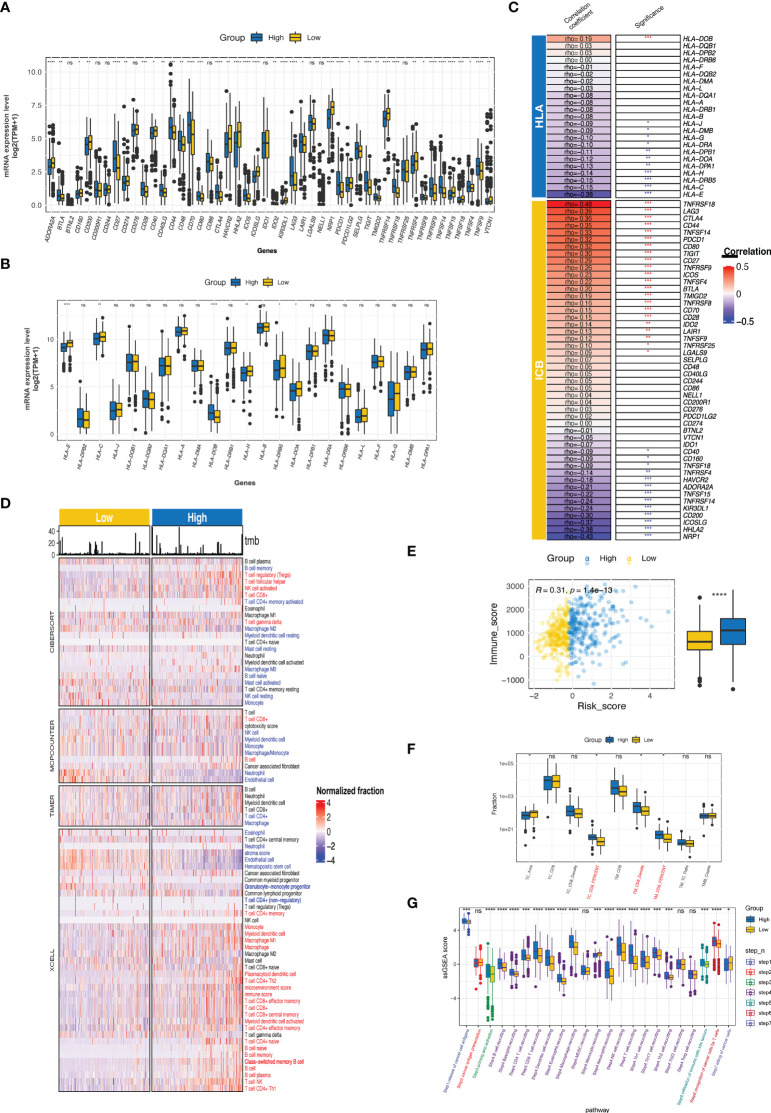
Immune phenotypes and tumor microenvironment related to CTR score. **(A)** Comparison of the expression level of immune checkpoints between high and low claudin-TME related (CTR) groups. **(B)** Analysis of the difference in the expression level of human leukocyte antigen (HLA) family genes between high and low CTR groups. **(C)** Correlation between CTR score and the expression level of immune checkpoints or HLA family genes. The asterisks indicate a significant statistical p value, and those colored with red and blue indicate positive and negative correlation with CTR score, respectively. **(D)** Comprehensive analysis of the difference in the tumor-infiltrated immune cells (TIICs) between high and low CTR groups using multiple algorithms (CIBERSOFT, MCPCOUNTER, TIMER, and XCELL). TIICs with blue color were significantly more enriched in the low CTR group; whereas, those with red color were more enriched in the high CTR group (p< 0.05). **(E)** Correlation between CTR score and immune score. **(F)** Difference in the CD8 + T cells between high and low CTR groups in an integration cohort with CheckMate-009, 010 and 025 cohorts. **(G)** Differences in the activity of seven steps of cancer-immunity cycle between two CTR groups. *p< 0.05, **p< 0.01, ***p< 0.001, ****p< 0.0001, ns, not significant; HLA, human leukocyte antigen; TC, tumor center; TM, tumor margin; TPM, transcript per million; ssGSEA, single-sample gene set enrichment analysis.

The CTR score was positively and negatively correlated with the expression of 21 and 11 immune checkpoints, respectively; significant associations were found with tumor necrosis factor receptor superfamily member 18 (TNFRSF18), lymphocyte activation gene 3 (LAG3), cytotoxic T-lymphocyte associated protein 4 (CTLA4), TNF superfamily member 14 (TNFSF14), programmed cell death protein 1 (PDCD1, PD-1), and T cell immunoreceptor with Ig and ITIM domains (TIGIT) (**Figures 9A, C**). Similar to the difference between cluster 1 and cluster 2 in MHC, the high-risk group had significantly lower expression levels of human leukocyte antigens (HLA) family genes (especially HLA-E), which may hinder the antigen presentation process (**Figures 9B, C**). Meanwhile, notable heterogeneity in the TIICs level was observed between the high and low CTR groups (**Figure 9D**). Using various algorithms, a concordant difference was identified in CD8 T cells, which was enriched in the high CTR group. A significantly higher immune score in the high CTR group was revealed by XCELL, which was also validated by the ESTIMATE algorithm (R = 0.29, p< 0.0001, **Figure 9E**). An immunohistochemistry (IHC) test of the ccRCC samples from the CheckMate studies showed a significantly higher level of CD8 + tumor cell (TC) ratio and CD8 + tumor margin (TM) density, which were found in the high CTR group, supporting the positive correlation between CTR score and CD8 T cells found using the algorithms (**Figure 9F**). Furthermore, the CTR score was associated with increased activity in all the steps of the cancer-immunity cycle, with the exception of step 2 (cancer antigen presentation, **Figure 9G**).

### 3.9 Construction of a nomogram by integrating the CTR score and clinicopathologic variables

**Figure 10:**
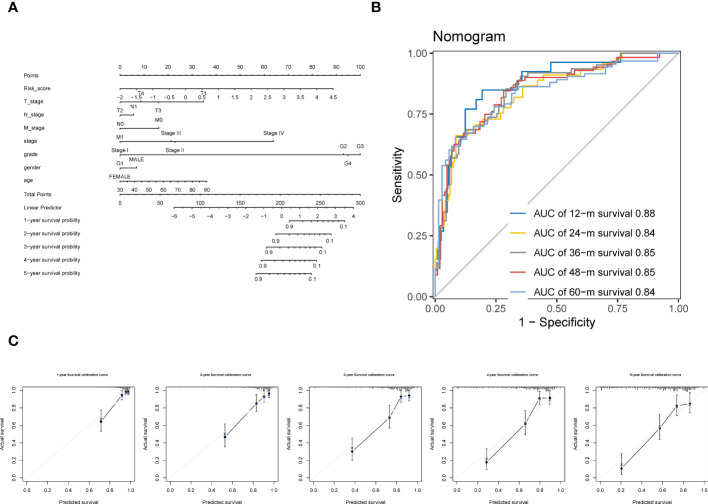
Construction of a Nomogram using integration CTR score and clinicopathologic variables. **(A)** claudin-TME related (CTR) score integrated with T (tumor stage), N (node stage), M (metastasis stage), neoplasm histologic grade, neoplasm disease stage, sex, and age to develop a Nomogram in ccRCC. **(B)** Time-dependent receiver operating characteristic (ROC) curves of the Nomogram. **(C)** Calibration curve for the prediction of overall survival (OS) at 1–5 year.

By incorporating the clinicopathologic variables (including T, N, M, neoplasm histologic grade, neoplasm disease stage, sex, and age) with the CTR score, a novel nomogram was established (**Figure 10A**). The ROC curve showed that the AUC values for predicting survival at 1, 2, 3, 4 and 5 years of this nomogram were 0.88, 0.84, 0.85, 0.85, and 0.84, respectively (**Figure 10B**). In addition, the 1–5 year calibration curves showed outstanding agreement between the actual observations and nomogram prediction (**Figure 10C**).

### 3.10 Estimation of treatment response correlated with CTR score

**Figure 11:**
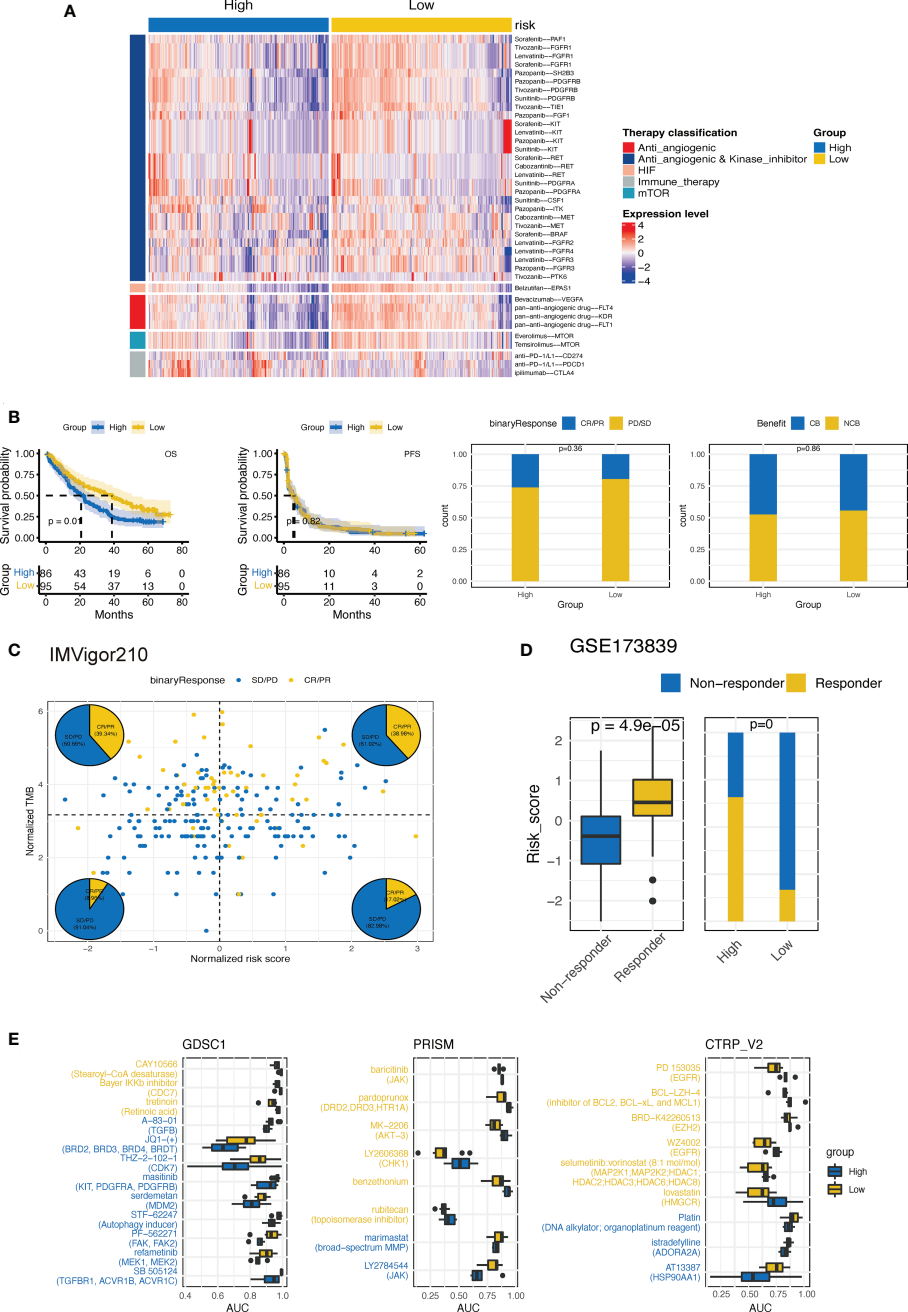
Estimation of treatment response correlated with CTR score. **(A)** Heatmap of the expression of ccRCC-related drug-target genes screened from the Drugbank database in high and low claudin-TME related (CTR) groups. **(B)** Kaplan–Meier curves and the difference in response rate between high and low CTR scores in nivolumab-treated ccRCC patients from CheckMate-009, 010 and 025 cohorts. **(C)** Response analysis with tumor mutational burden (TMB) and CTR score stratification in IMvigor210 cohort. **(D)** Comparison of the CTR score between responders and nonresponders and the response rate between high and low CTR score groups from the GSE173839 cohort. **(E)** Three drug sensitivity databases (CTRP-V2, PRISM, and GDSC1) were used to identify the sensitivity of high and low CTR group cell line subsets to specific agents. Agents with lower area under the curve (AUC) values on the x-axis of boxplots had a greater drug sensitivity, and those colored with blue had a higher sensitivity in the high CTR group.

We then explored whether the established CTR score could stratify ccRCC patients into different systemic treatments with differed efficacies. First, we evaluated the difference in the expression of ccRCC-related therapeutic target genes between the high and low CTR groups. Intriguingly, the majority of targeted genes of these selected drugs, most of which were antiangiogenic and kinase inhibitors, were overexpressed in the low CTR group (**Figure 11A**). The low-risk group showed significant overexpression of both the markers of pan-antiangiogenic drug, such as vascular endothelial growth factor receptor 1 (VEGFR1) (Fms related receptor tyrosine kinase 1 (FLT1)), VEGFR2 (kinase insert domain receptor (KDR)), and VEGFR3 (FLT4), and specific markers, including VEGFA, colony stimulating factor 1 (CSF1), SH2B adaptor protein (SH2B3), fibroblast growth factor receptor (FGFR1/2/3/4), B-Raf proto-oncogene (BRAF), Raf-1 proto-oncogene (RAF1), and tyrosine kinase with immunoglobulin-like and EGF-like domains 1 (TIE1). The expression of endothelial PAS domain protein 1 (EPAS1), the main target of belzutifan, which was a novel breakthrough HIF inhibitor, was also significantly upregulated in the low-risk group. However, the targets of ICIs, including CD274, PDCD1, and CTLA4, were upregulated in the high CTR group. Hence, we investigated whether the CTR score could distinguish patients who had improved clinical benefits with ICIs. In nivolumab-treated ccRCC patients, there was no significant difference in the objective response rate (complete response (CR) + partial response (PR)) or clinical benefit rate between the high and low CTR groups (**Figure 11B**). Even though the group with a low CTR had a longer overall survival (OS), this may be attributed more to the model’s prognostic prediction function than to the increased sensitivity to nivolumab. In the IMVigor210 cohort, we found that CTR may not function as an effective ICI biomarker compared to TMB; however, in the TMB-low subgroups, there was a higher ratio of responders in the high CTR group (**Figure 11C**). In the GSE173839 cohort, patients who responded to ICI combination therapy had significantly higher CTR scores, and more responders were present in the high CTR group (**Figure 11D**). Furthermore, we utilized three drug response databases, GDSC1, PRISM, and CTRP-v2, to further identify CTR score-related therapeutic agents in ccRCC (**Figure 11E**). Among these agents, more immunity-related drugs (such as TGF β-related and janus kinase (JAK) inhibitors) were identified in the high CTR group, while more tyrosine kinase inhibitors (EGFR-related) were identified in the low CTR group.

## 4 Discussion

Interestingly, in our study, both the cluster 1 and high CTR groups, which were associated with worse outcomes, were characterized by claudin-low features. The specific genomic, TME, and clinicopathological characteristics of claudin-low ccRCC phenotype remain unclear. It is a novel molecular subtype of breast and bladder cancer, with a prominent role in the downregulation of cell-cell adhesion genes and overexpression of epithelial–mesenchymal transition (EMT) genes and stem cell-related genes (14). In our study, we also found that cluster 1 and high CTR groups displayed notable stem cell-like features (increased mRNAsi score), but no substantial upregulation of EMT-related genes. Furthermore, in concordance with our results, other studies have shown that claudin-low tumors were enriched in immune and stromal cell infiltration (16, 41), especially a high abundance of regulatory T cells, revealing an active immunosuppression patten (43). These findings confirm our hypothesis regarding the existence of the claudin-low subtype in ccRCC, which shares similar TME and stemness characteristics with breast and bladder cancer.

However, while comprehensively discovering the separation of two claudin clusters in five public datasets and local ccRCC patients, we cannot directly apply its prognostic stratification function without first developing a predictor or risk model. Subsequently, by employing a series of algorithms and integrating the 12 identified CTR-related genes, we created a prognostic prediction signature with both stable and robust accuracy that surpassed a number of previously defined ccRCC risk models. Moreover, it can be applied to various other types of cancers, especially pan-kidney cancers (KIRC, KICH, and KIRP). The CTR score also outperformed the traditional clinicopathological factors, including clinical and histological stages, and both the clinicopathological and genomic features supported the prognostic prediction value of the signature. More known poor prognosis-related GAs, especially *BAP1* (44) and *SETD2* (45), were identified in the high CTR group. Although we found more GAs with different prevalence in the high CTR group, there was no significant correlation between CTR score and TMB, which may be attributed to the fact that ccRCC has a comprehensively modest TMB level among solid tumors (46). Meanwhile, higher chromosomal instability (CIN), characterized by the enrichment of CNV alterations, was identified in the high CTR group. Due to the increasing frequency of CNV during cancer cell proliferation, CIN-positive tumors exhibit greater intratumor heterogeneity and are more prone to develop therapeutic resistance due to their enhanced capacity to adapt to selection pressures (47). We previously found that increasing CIN drives invasion and metastasis in ccRCC (48), and in this study, some of these differed CNVs were found to be related to the progression of ccRCC. The TRACERx Renal project identified that deletion of 9p21, including the loss of tumor suppressor cyclin-dependent kinase inhibitor 2A (CDKN2A) is a pivotal event driving the metastasis of ccRCC and related death (49). Contrarily, CIN may also function as an enhancer of cancer immunology; induction of CIN promotes the upregulation of pro-inflammation genes, natural killer (NK) cell-activating ligands, and cytokine secretion (50); moreover, a combination of agents that promote CIN and ICI could inhibit tumor growth (51). David et al. also revealed that CD8+ T cell-infiltrated ccRCC tumors are more enriched with deletions at 9p21 than those with noninfiltrated ones. This was consistent with our findings that the high CTR group with notable CIN may have higher tumor immunity and an associated elevated sensitivity to ICIs.

Although there was a strong correlation between the number of CD8 T cells and the CTR score, this did not translate to a phenotype with greater therapeutic benefits to nivolumab monotherapy. This was consistent with a previous finding, which showed that there was no significant correlation between baseline CD8 T cells and response to ICI in ccRCC (39). However, in TMB-low patients from the IMVigor210 cohort or the combination therapy cohort (GSE173839), the high CTR group presented with more responders to ICIs. This may be ascribed to the differences in tumor immunology and genomic characteristics between ccRCC and other malignancies such as bladder or lung cancer for which conventional immunotherapy knowledge is available (52). Furthermore, significant overexpression of VEGFR1, VEGFR2, PDGFRB, and VEGFR3 was found in the low-risk group. Benoit et al. found that the expression level of these markers was associated with survival benefit in ccRCC patients treated with sunitinib, which is the standard first-line treatment (53). In addition to antiangiogenic and multiple kinase inhibitors, the low CTR group was also more sensitive to mTOR or HIF-2α inhibitors. Belzutifan is a selective inhibitor targeting HIF-2α, which has been approved by the US FDA to treat RCC with VHL disease and has a promising objective response rate (ORR) of 49% (54). In phase I trial with heavily pretreated ccRCC patients (NCT02974738), it also showed an effective antitumor efficacy with ORR of 25% and progression-free survival (PFS) of 14.5 months (55). The differential expression of TEK, which is a hub gene in this signature, may account for this disparity in hypoxia and sensitivity to associated therapeutic agents between the CTR groups.

In conclusion, the current study provided comprehensive insights into understanding the claudin-low phenotype in ccRCC and highlighted its association with the TME feature. To enhance its clinical utility, we further developed a prognostic prediction signature based on the interaction between claudin-low phenotype and TME, which could accurately predict the outcomes of patients with ccRCC and other histological forms of kidney cancer. Moreover, it was also an effective biomarker in treatment stratification, including targeted therapy and ICIs. These features may contribute to better personalizing management of patients with ccRCC.

## Data availability statement

The data presented in the study are deposited in the China National Center for Bioinformation/National Genomics Data Center of China (https://ngdc.cncb.ac.cn/gsahuman/) accession number HRA003416.

## Ethics statement

The studies involving human participants were reviewed and approved by Biomedical Research Ethics Committee of Peking University First Hospital. The patients/participants provided their written informed consent to participate in this study.

## Author contributions

CZ: conception and design, literature research, clinical studies, experimental studies, data analysis, manuscript editing, manuscript preparation. YL and JQ: data acquisition and analysis. ZZ: literature research, experimental studies, data analysis, statistical analysis. CH: literature research and data acquisition. YG: conception and design, manuscript preparation and editing; ZH and LZ: definition of intellectual content, manuscript review. All authors contributed to the article and approved the submitted version.

## Funding

The present study was funded by The National Key R&D Program of China (grant no. 2019YFA0906001) and National Natural Science Foundation of China (No. 82273347).

## Conflict of interest

The authors declare that the research was conducted in the absence of any commercial or financial relationships that could be construed as a potential conflict of interest.

The reviewer NZ declared a shared affiliation with the authors to the handling editor at time of review.

## Publisher’s note

All claims expressed in this article are solely those of the authors and do not necessarily represent those of their affiliated organizations, or those of the publisher, the editors and the reviewers. Any product that may be evaluated in this article, or claim that may be made by its manufacturer, is not guaranteed or endorsed by the publisher.

## Supplementary material

Supplementary Figure 1 The expression feature and prognostic function of claudin family genes in kidney cancer. **(A)** The difference in the expression level of each claudin gene between tumor and normal tissues in kidney renal clear cell carcinoma (KIRC) (sample size, cancer vs normal: 530 vs 72), kidney renal papillary cell carcinoma (KIRP) (288 vs 32) and kidney chromophobe (KICH) (66 vs 25). **(B)** Forest plots illustrating univariate analyses for overall survival stratification with each claudin gene. CLDN13 and CLDN21 are not included because no valid expression data is available; *p< 0.05, **p< 0.01, ***p< 0.001, ****p< 0.0001, ns: not significant. KIRC: kidney renal clear cell carcinoma; KIRP: Kidney renal papillary cell carcinoma; KICH: kidney chromophobe.

Supplementary Figure 2 Validation of the expression feature of claudin genes in other dataset and local ccRCC samples. **(A)** Silhouette clustering analysis in GSE40435, GSE53757, International Cancer Genome Consortium (ICGC) datasets, and local ccRCC samples; **(B)**.
