# Supplementary figures and images for: Identification of a claudin-low subtype in clear cell renal cell carcinoma with implications for the evaluation of clinical outcomes and treatment efficacy
Source: Front Immunol. 2022 Nov 21;13:1020729. doi: 10.3389/fimmu.2022.1020729 (PMC9719924; doi:10.3389/fimmu.2022.1020729)

A

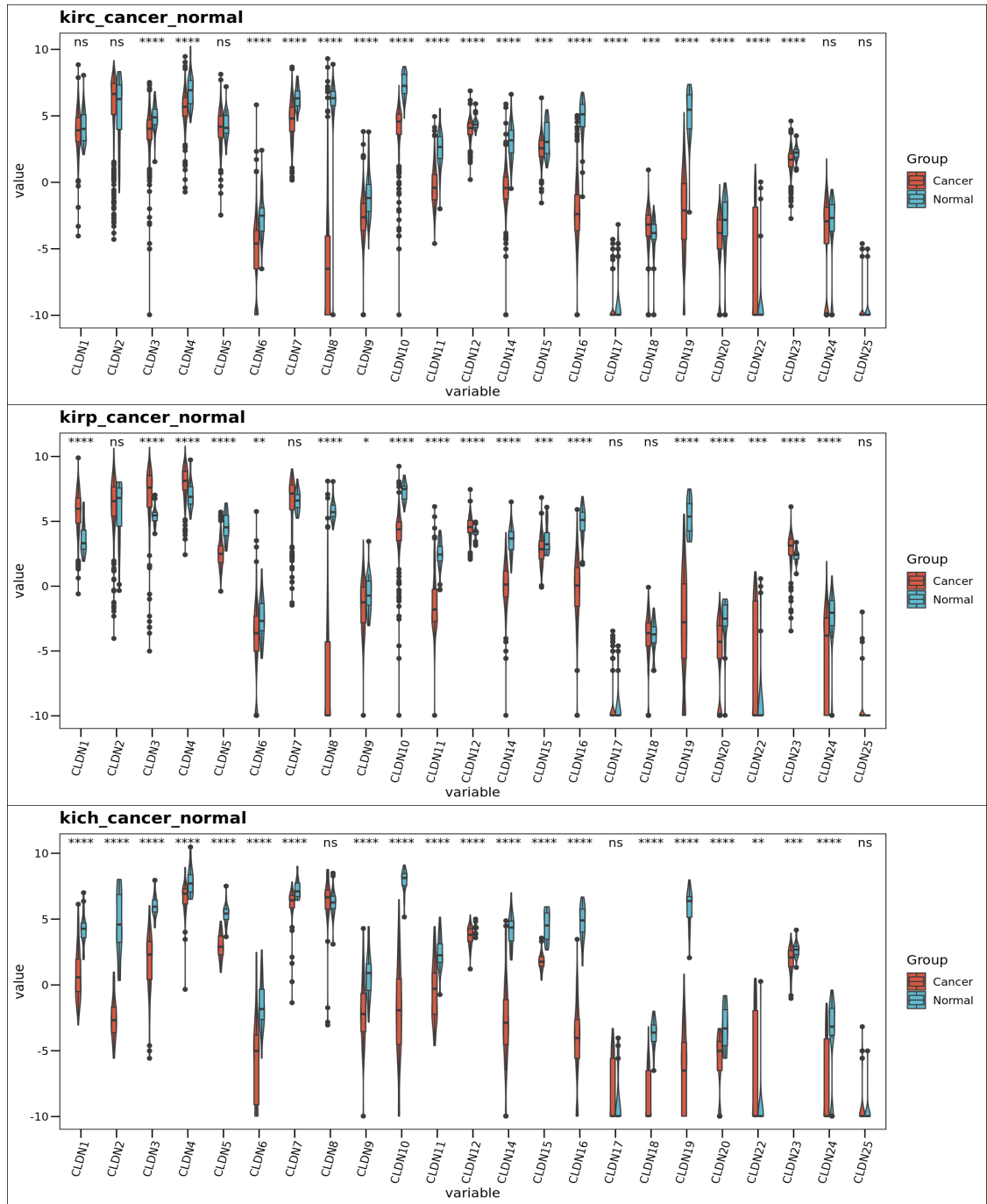

B

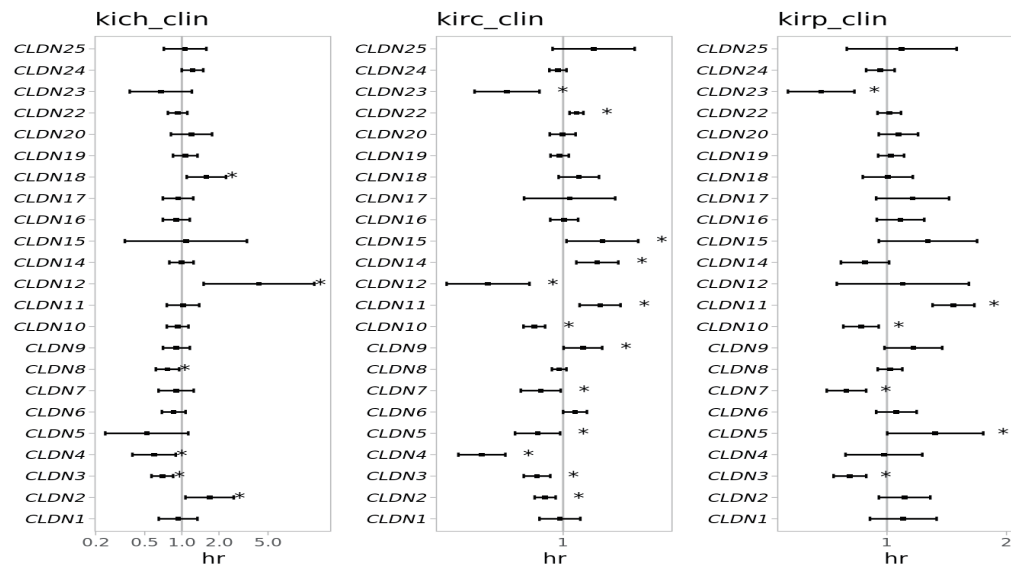

Supplement: Supplementary Figure 1 — The expression feature and prognostic function of claudin family genes in kidney cancer. (A) The difference in the expression level of each claudin gene between tumor and normal tissues in kidney renal clear cell carcinoma (KIRC) (sample size, cancer vs normal: 530 vs 72), kidney renal papillary cell carcinoma (KIRP) (288 vs 32) and kidney chromophobe (KICH) (66 vs 25). (B) Forest plots illustrating univariate analyses for overall survival stratification with each claudin gene. CLDN13 and CLDN21 are not included because no valid expression data is available; *p< 0.05, **p< 0.01, ***p< 0.001, ****p< 0.0001, ns: not significant. KIRC: kidney renal clear cell carcinoma; KIRP: Kidney renal papillary cell carcinoma; KICH: kidney chromophobe. [file DataSheet_1.pdf]

A

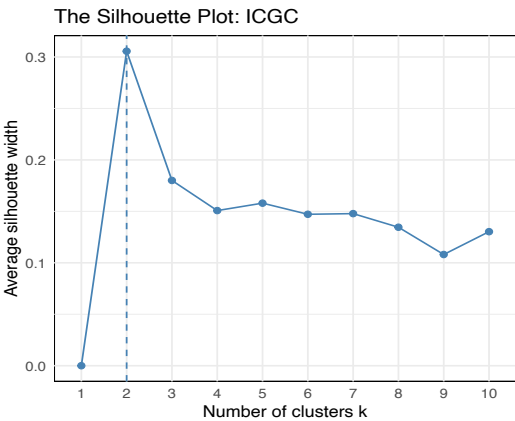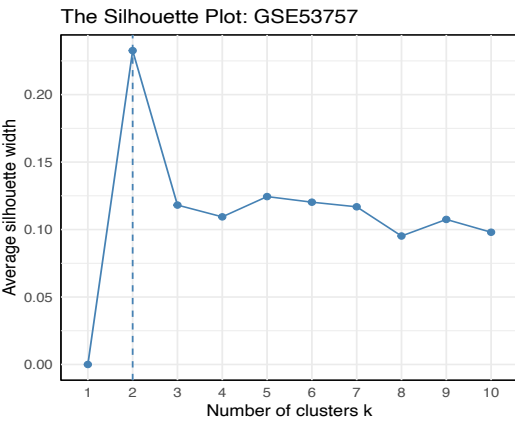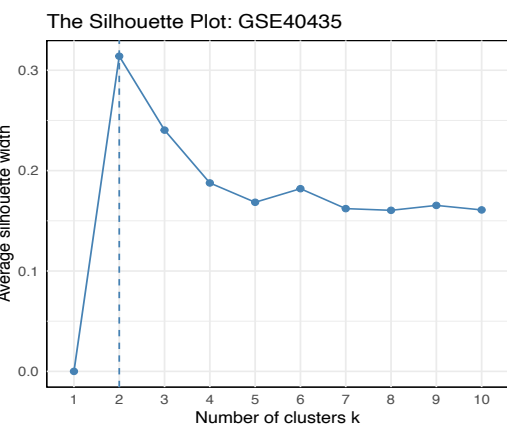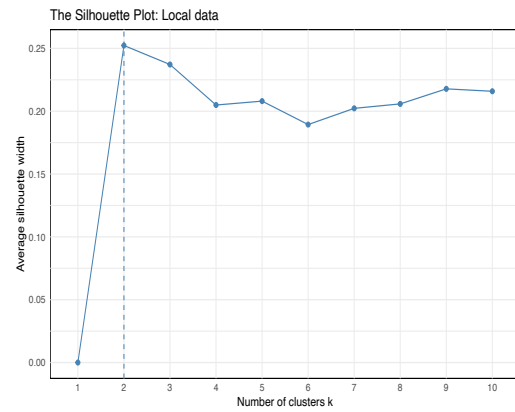

B

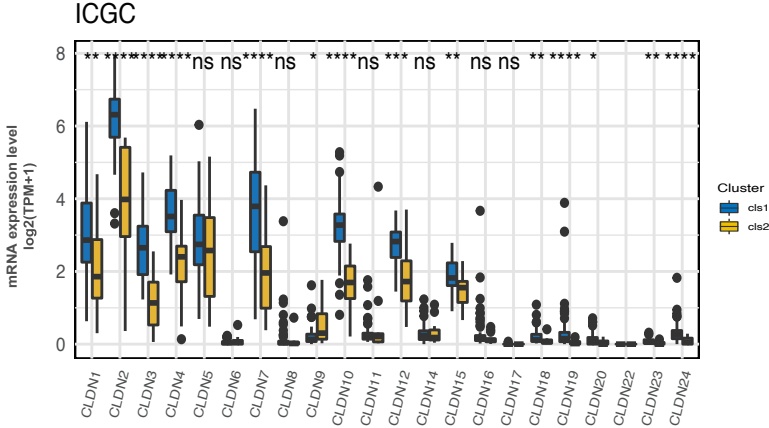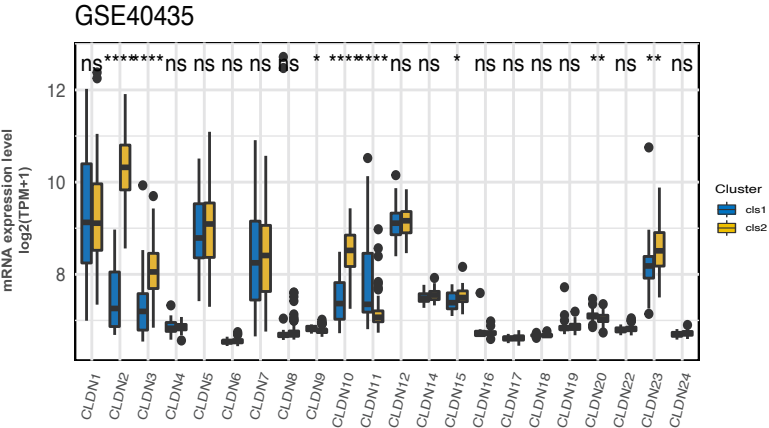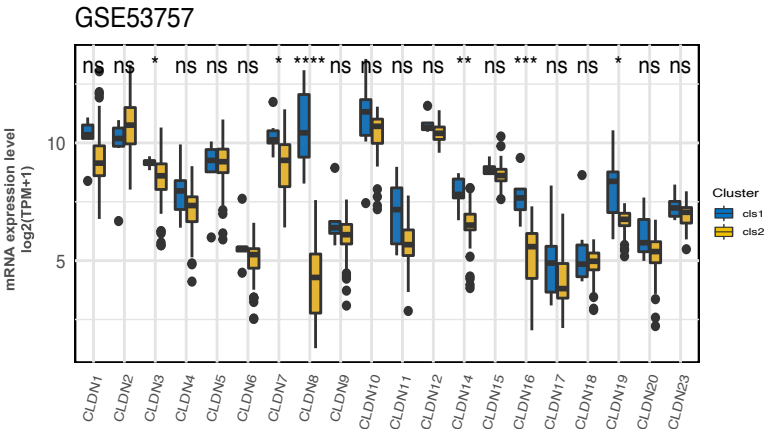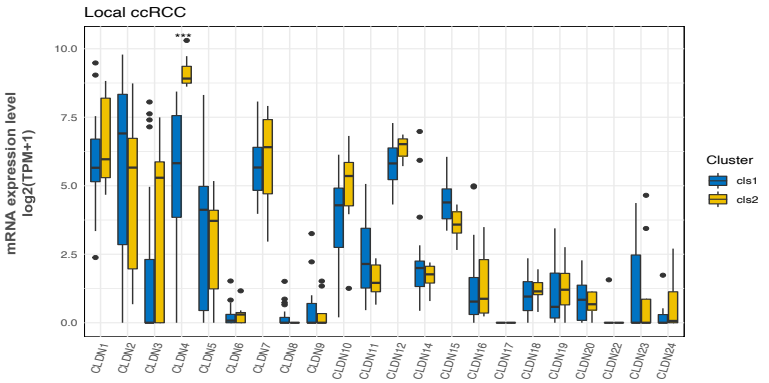

Supplement: Supplementary Figure 2 — Validation of the expression feature of claudin genes in other dataset and local ccRCC samples. (A) Silhouette clustering analysis in GSE40435, GSE53757, International Cancer Genome Consortium (ICGC) datasets, and local ccRCC samples; (B). [file DataSheet_2.pdf]
